# Enhanced Efficacy of Thalidomide‐Based Cocktail With Transarterial Chemoembolization in the Management of Unresectable Hepatocellular Carcinoma: A Multi‐Center Cohort Study

**DOI:** 10.1002/mco2.71116

**Published:** 2026-10-31

**Authors:** Jinzhi Xu, Li Song, Yifan Kong, Jie Li, Jian Zhang, Tao Ye, Yongqiang Liu, Xin Yin, Biwei Yang, Zhiying Zhao, Peixin Huang, Yi Chen, Matteo Jiang, Ningling Ge, Faiz Wali, Wenhao Hu, Guoqing Zhu, Jinglin Xia

**Affiliations:** ^1^ National Medical Center and National Clinical Research Center for Interventional Medicine, Liver Cancer Institute, Zhongshan Hospital Fudan University Shanghai China; ^2^ Department of Hematology, Shanghai Geriatric Medical Center Zhongshan Hospital Fudan University Minhang Campus Shanghai China; ^3^ Department of Infectious Diseases Shanghai Sixth People's Hospital Affiliated to Shanghai Jiao Tong University School of Medicine Shanghai China; ^4^ Zhejiang Key Laboratory of Intelligent Cancer Biomarker Discovery and Translation The First Affiliated Hospital of Wenzhou Medical University Wenzhou Zhejiang China; ^5^ Department of Oncology Minhang Hospital Fudan University Shanghai China; ^6^ Department of Radiology Kecheng People's Hospital Quzhou Zhejiang China; ^7^ Department of Interventional Radiology The First Affiliated Hospital of Wenzhou Medical University Wenzhou Zhejiang China

**Keywords:** hepatocellular carcinoma (HCC), metronomic chemotherapy, TCC cocktail, transarterial chemoembolization (TACE), uracil analogues

## Abstract

This retrospective study was designed to evaluate the safety and efficacy of adding a thalidomide‐based cocktail (the TCC cocktail—comprising thalidomide, carmofur, and compound mylabris capsule) to transarterial chemoembolization (TACE) for unresectable hepatocellular carcinoma (HCC) in real‐world practice. Data from 433 unresectable HCC patients treated with TACE at four hospitals were collected (from March 2014 to May 2018, followed up to July 2023) and overall survival (OS), overall response rate (ORR), disease control rate (DCR), and adverse events (AEs) were analyzed. After 1: 2 propensity score matching (PSM), 114 patients in the cocktail group (TACE + TCC) were compared with 228 patients in the control group (TACE alone). The median OS of the cocktail group was significantly better than that of the control group (*p* < 0.001; 42.5 vs. 12.5 months) after PSM. ORR (29.8% vs. 21.1%, *p* = 0.081) and DCR (79.8% vs. 61.8%, *p* < 0.001) at 6 months were also better in the cocktail group after PSM. Moreover, no life‐threatening AEs (grade 4 or 5) occurred in either group. In this retrospective cohort, the addition of the TCC cocktail to TACE was associated with improved survival and tumor response, with a manageable safety profile.

## Graphical abstract

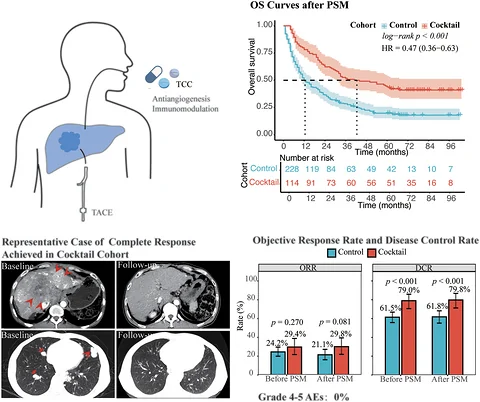

## 1 Introduction

Hepatocellular carcinoma (HCC) is one of the most common malignancies worldwide [1, 2]. Due to its insidious onset, approximately 70% of HCC patients are diagnosed at an advanced stage according to Barcelona Clinic Liver Cancer (BCLC) staging [3], thus losing the opportunity for curative surgical resection. For these patients, transarterial chemoembolization (TACE) remains the primary locoregional treatment option [4, 5], but it often leads to incomplete tumor response and disease progression, necessitating repeated procedures [6]. Over the past decade, systemic therapies have evolved significantly. Sorafenib established the role of molecular targeted therapy (MTT) in advanced HCC [7, 8], and more recently, immunotherapy‐based regimens (e.g., atezolizumab–bevacizumab) have further improved survival [9, 10, 11]. Nonetheless, these approaches are limited by adverse events, variable efficacy, lack of predictive biomarkers, and high cost [12, 13, 14].

Combining TACE with systemic agents has shown mixed results [14]. While some studies report improved progression‐free survival and objective response rates, a consistent overall survival benefit remains elusive [15]. Moreover, the addition of MTT or immunotherapy to TACE increases the incidence of grade 3–4 adverse events [16, 17]. These uncertainties highlight the need for alternative combination strategies.

Inspired by the success of cocktail therapy in AIDS [18], we developed a triple‐drug regimen—thalidomide, carmofur, and compound mylabris capsule (CMC)—termed the TCC cocktail [19]. Each component has distinct anti‐tumor mechanisms: thalidomide exerts anti‐angiogenic and immunomodulatory effects [20], carmofur inhibits DNA/RNA synthesis [21, 22], and cantharidin (the active ingredient of CMC [23]) induces apoptosis and DNA damage [24, 25].

Building on these findings, our previous single‐center retrospective study of 92 unresectable HCC patients demonstrated that TCC significantly improved 2‐year OS rates in patients treated with TACE (61.6% vs. 21.8%, *p* = 0.004) [19]. Further mechanistic exploration revealed that TCC could target the SAMD4B‐APOA2‐PD‐L1 axis, thereby alleviating tumor‐related immune suppression [26]. However, these earlier findings were derived from a single‐center study with limited sample size, and whether the survival benefit can be replicated in a multicenter real‐world setting remains unknown. Accordingly, the present multicenter retrospective study was designed to validate the effectiveness and safety of TCC combined with TACE for unresectable HCC.

## 2 Results

### 2.1 Baseline Characteristics of Patients

In total, 545 HCC patients who received initial TACE were screened, with 112 being excluded according to the study protocols (shown in Figure 1). The whole cohort consisted of 119 patients in the cocktail group and 314 in the control group. Before PSM, several baseline characteristics were imbalanced between the cocktail and control groups, with absolute standardized mean differences (SMDs) exceeding 0.1: age (0.150), hepatitis history (0.203), Child–Pugh grade (0.274), BCLC stage (0.316), extrahepatic metastasis (0.202), ECOG PS (0.174), hemoglobin (0.248), and white blood cell count (0.119).

**FIGURE 1:**
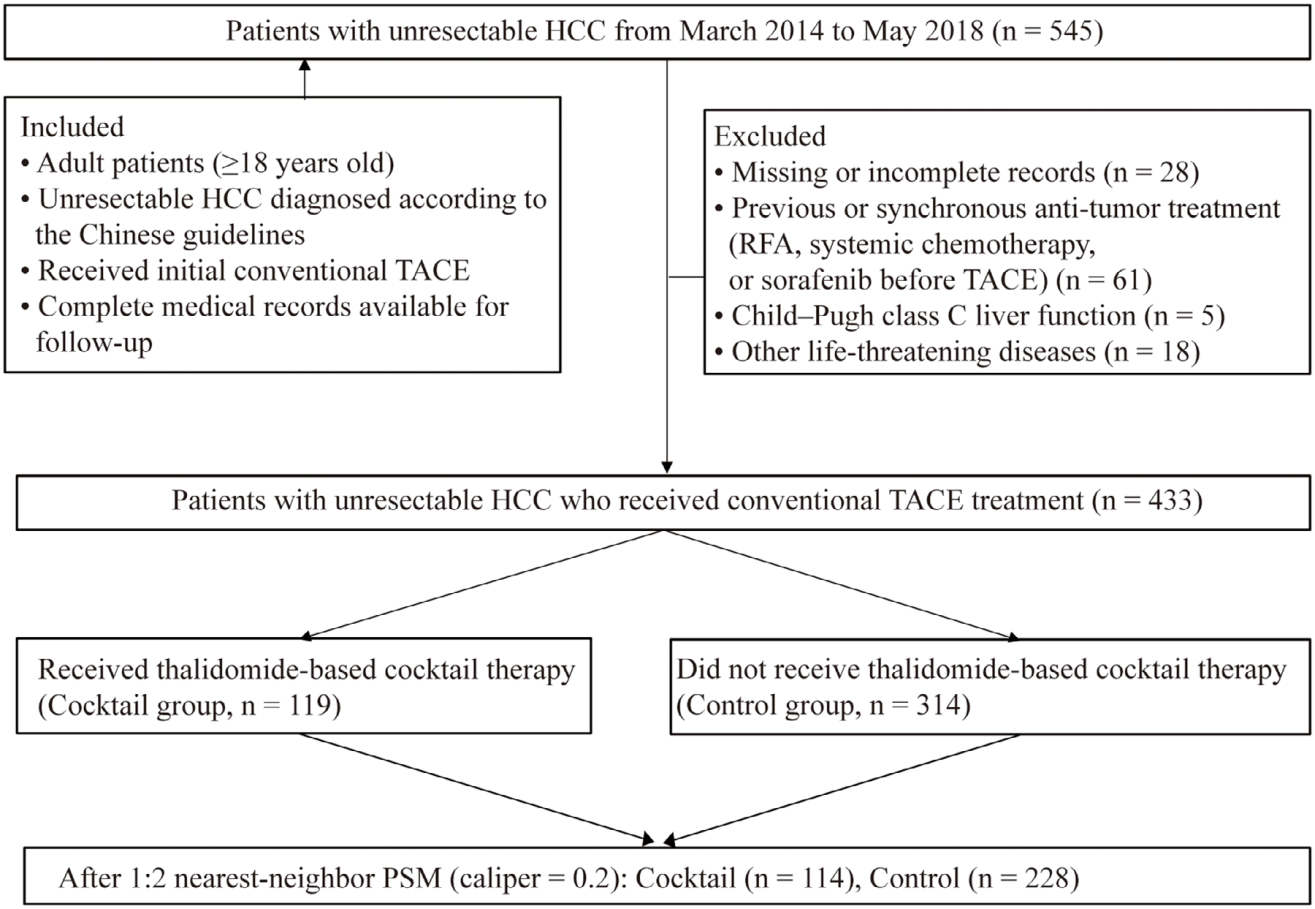
Flowchart of patient selection and construction of pre‐PSM and post‐PSM cohorts. A total of 545 patients with hepatocellular carcinoma who received initial transarterial chemoembolization (TACE) were screened. According to the study protocol, 112 patients were excluded. The final pre‐matching cohort comprised 433 patients: 119 in the cocktail group and 314 in the control group. After 1:2 nearest‐neighbor propensity score matching, 114 patients remained in the cocktail group and 228 in the control group. TACE, transarterial chemoembolization.

After 1:2 nearest‐neighbor matching, the cohort sizes were reduced to 114 in the cocktail group and 228 in the control group. Balance was assessed using the *bal.tab()* function in the R package *cobalt*, and all covariates had adjusted differences below 0.1 after matching, indicating good balance between the two groups. The distribution of propensity scores before and after matching is shown in Figure 2A, and the Love plot of SMDs is shown in Figure 2B. The baseline characteristics of the whole cohort and the well‐matched cohort are listed in Table 1.

**FIGURE 2:**
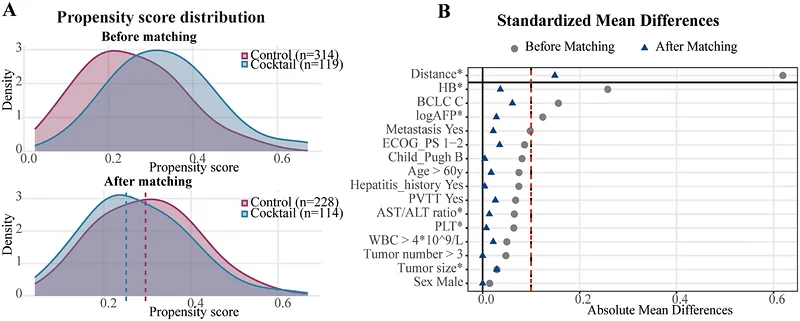
Distribution of propensity scores before and after PSM. (A) Distribution of propensity scores before and after matching. **(B)** Love plot of absolute standardized mean differences (SMDs) before and after matching. The vertical dashed line indicates an SMD of 0.1. After matching, all covariates had absolute SMDs < 0.1, indicating adequate balance between groups. PSM, propensity score matching; SMD, standardized mean difference. ^*^ indicates continuous variables.

**TABLE 1:** Baseline clinical characteristics of patients in the control and cocktail group before and after PSM.

Variables	Before PSM Mean ± SD/*N* (%)			After PSM Mean ± SD/*N* (%)		
	Control (314)	Cocktail (119)	|SMD|	Control (228)	Cocktail (114)	|SMD|
Age (years)	60.92 ± 12.53	56.00 ± 11.13	0.150	59.61 ± 11.92	56.47 ±10.95	0.018
Sex			0.038			<0.001
Female	60 (19.1)	21 (17.6)		40 (17.5)	20 (17.5)	
Male	254 (80.9)	98 (82.4)		188 (82.5)	94 (82.5)	
Hepatitis history			0.203			0.004
No	63 (20.1)	15 (12.6)		29 (12.7)	15 (13.2)	
Yes	251 (79.9)	104 (87.4)		199 (87.3)	99 (86.8)	
Child–Pugh grade			0.274			0.004
A	270 (86.0)	112 (94.1)		215 (94.3)	108 (94.7)	
B	44 (14.0)	7 (5.9)		13 (5.7)	6 (5.3)	
ECOG‐PS			0.174			0.035
0	167 (53.2)	53 (44.5)		114 (50.0)	53 (46.5)	
1–2	147 (46.8)	66 (55.5)		114 (50.0)	61 (53.5)	
BCLC stage			0.316			0.061
B	181 (57.6)	50 (42.0)		114 (50.0)	50 (43.9)	
C	133 (42.4)	69 (58.0)		114 (50.0)	64 (56.1)	
Tumor diameter (mm)	68.11 ± 43.20	69.43 ± 44.61	0.017	65.91 ± 43.01	67.21 ± 42.73	0.029
Number of tumors			0.101			<0.001
≤ 3	118 (37.6)	39 (32.8)		76 (33.3)	38 (33.3)	
> 3	196 (62.4)	80 (67.2)		152 (66.7)	76 (66.7)	
PVTT			0.143			0.026
No	214 (68.2)	73 (61.3)		146 (64.0)	70 (61.4)	
Yes	100 (31.8)	46 (38.7)		82 (36.0)	44 (38.6)	
Metastasis			0.202			0.022
No	205 (65.3)	66 (55.5)		137 (60.1)	66 (57.9)	
Yes	109 (34.7)	53 (44.5)		91 (39.9)	48 (42.1)	
Hb (g/L)	129.75 ± 20.69	134.71 ± 19.19	0.248	134.34 ± 19.06	135.04 ± 18.72	0.037
WBC (10^9^/L)	5.66 ± 2.50	6.17 ± 2.66	0.119	5.58 ± 2.35	6.15 ± 2.7	0.022
PLT (10^9^/L)	165.74 ± 93.37	161.06 ± 72.8	0.056	159.01 ± 86.61	158.45 ± 71.81	0.008
ALT (U/L)	50.44 ± 62.40	48.93 ± 38.39	NA	52.13 ± 68.53	49.88 ± 38.87	NA
AST (U/L)	75.96 ± 83.97	74.73 ± 81.12	NA	77.08 ± 91.68	72.68 ± 80.82	NA
AST/ALT			0.085			0.014
AFP (ng/mL)	7431.66 ± 16877.28	5991.96 ± 15918.14	NA	7115.06 ± 16993.06	5723.37 ± 15393.84	NA
Log AFP			0.269			0.028

*Note*: Data are expressed as means ± SD or number (percentages). SMD absolute value < 0.1 is considered good balance, < 0.2 acceptable balance, and > 0.2 meaningful imbalance.

Abbreviations: AFP, alpha fetoprotein; ALT, alanine aminotransferase; AST, aspartate aminotransferase; BCLC, Barcelona Clinic Liver Cancer; Hb, hemoglobin; PLT, platelet; PSM, propensity score matching; PVTT, portal vein tumor thrombus; WBC, white blood cell.

### 2.2 Survival Analysis

Survival analyses were performed both before and after PSM to evaluate the prognostic impact of the cocktail regimen compared to the control regimen as shown in Table 2.

**TABLE 2:** Survival analysis comparing the control and cocktail groups before and after PSM.

Survival analysis	Control	Cocktail	*p*‐value
Before PSM			
Median OS (95% CI, months)	15.6 (11.9–18.7)	37.0 (30.3–67.9)	< 0.001^*^
1‐year survival rate (%, 95% CI)	48.7 (43.5–54.6)	78.2 (71.1–85.9)	
3‐year survival rate (%, 95% CI)	25.7 (21.3–31.1)	50.4 (42.2–60.3)	
5‐year survival rate (%, 95% CI)	17.5 (13.8–22.3)	42.0 (34.0–51.9)	
After PSM			
Median OS (95% CI, months)	12.5 (10.0–19.0)	42.5 (31.0–NR)	<0.001^*^
1‐year survival rate (%, 95% CI)	50 (43.9–56.9)	78.1 (70.8–86.0)	
3‐year survival rate (%, 95% CI)	28.5 (23.2–35.0)	51.8 (43.3–61.8)	
5‐year survival rate (%, 95% CI)	19.8 (15.2–25.7)	43.9 (35.6–54.0)	

* *p*‐values <0.05 were considered to be statistically significant.

Abbreviations: PSM, propensity score matching; CI, confidence intervals; NR, not reached; OS, overall survival.

#### 2.2.1 Survival Outcomes Before PSM

As a supportive analysis, overall survival was assessed before PSM. Patients in the cocktail group exhibited significantly longer median overall survival (OS) compared with the control group (37.0 months [95% CI: 30.3–67.9] vs. 15.6 months [95% CI: 11.9–18.7], log‐rank *p* < 0.001, Figure 3A). The 1‐, 3‐, and 5‐year survival rates were consistently higher in the cocktail group, with 1‐year rates of 78.2% (95% CI: 71.1–85.9) vs. 48.7% (95% CI: 43.5–54.6), 3‐year rates of 50.4% (95% CI: 42.2–60.3) vs. 25.7% (95% CI: 21.3–31.1), and 5‐year rates of 42.0% (95% CI: 34.0–51.9) vs. 17.5% (95% CI: 13.8–22.3).

**FIGURE 3:**
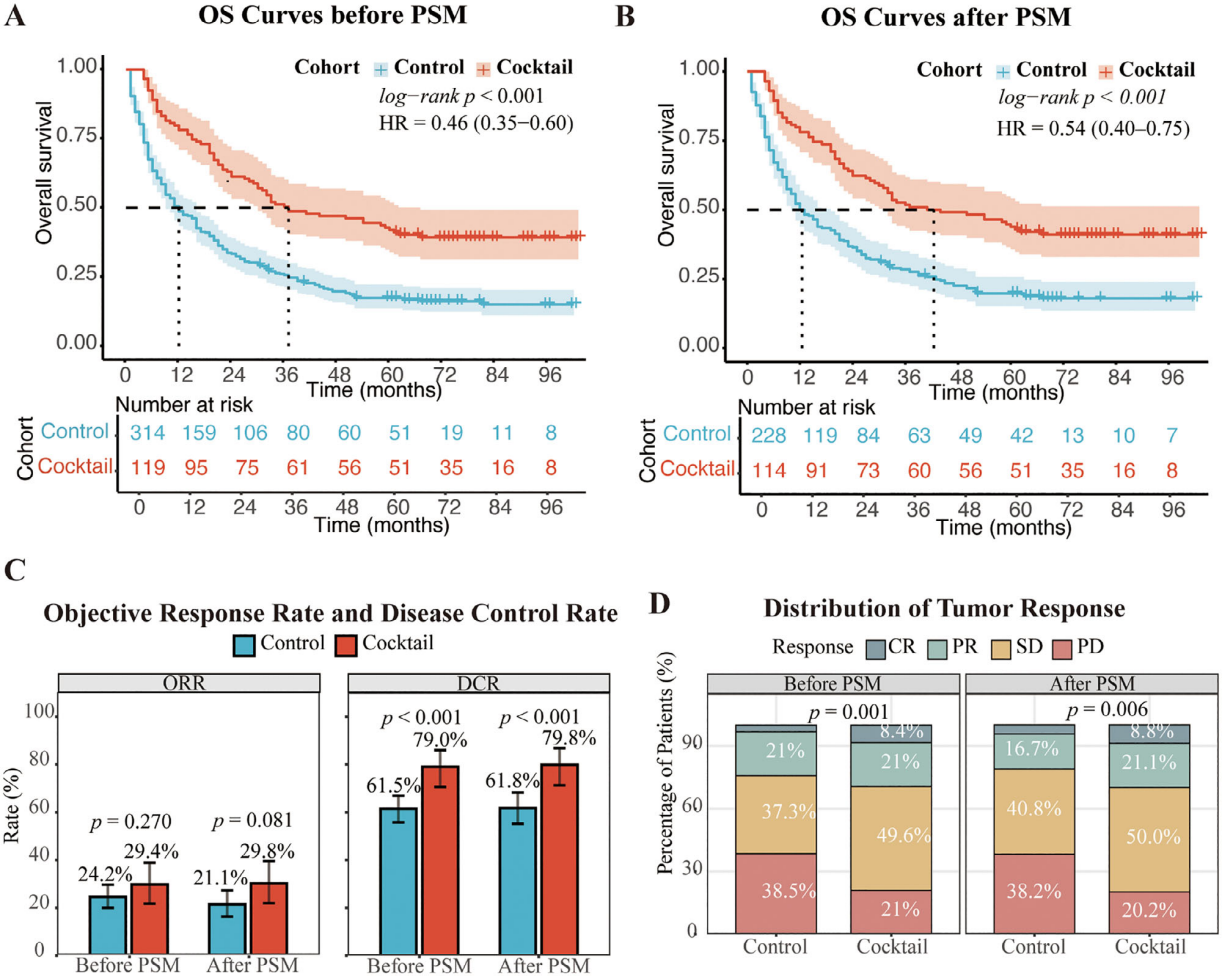
Survival analyses and treatment response before and after PSM. (A) Kaplan–Meier estimates of overall survival in the pre‐matching cohort (cocktail, *n* = 119; control, *n* = 314). **(B) **Kaplan–Meier estimates of overall survival in the post‐matching cohort (cocktail, *n* = 114; control, *n* = 228). *p* values in (A) and (B) are log‐rank *p* values. The hazard ratio (HR) and 95% confidence interval (CI) shown in (B) were derived from a treatment‐only Cox proportional hazards model with treatment group as the only covariate (HR = 0.54, 95% CI 0.40–0.75; *p* < 0.001). **(C) **Objective response rate (ORR) and disease control rate (DCR) before and after matching. **(D)** Distribution of complete response (CR), partial response (PR), stable disease (SD), and progressive disease (PD) before and after matching. ORR was defined as CR + PR; DCR was defined as CR + PR + SD. *p* values in (C) and (D) were calculated using Fisher's exact test and the chi‐square test, respectively. PSM, propensity score matching; OS, overall survival; HR, hazard ratio; CI, confidence interval; ORR, objective response rate; DCR, disease control rate; CR, complete response; PR, partial response; SD, stable disease; PD, progressive disease.

In the pre‐PSM cohort, univariable Cox analysis identified the cocktail regimen as a significant protective factor for OS (HR = 0.46, 95% CI 0.35–0.60, *p* < 0.001). Other significant factors included age > 60 years, male sex, Child–Pugh B, AFP > 20 ng/mL, low hemoglobin, elevated AST, larger tumor size, PVTT, and extrahepatic metastasis (all *p* < 0.05, Table S1). To adjust for potential confounders and avoid collinearity, we constructed a clinically driven multivariable main model including BCLC stage but excluding tumor size, PVTT, and extrahepatic metastasis. In the clinically driven multivariable main model (including BCLC stage, but excluding tumor size, PVTT, and metastasis, Figure S1A and Table S1), the cocktail regimen remained independently protective (adjusted HR = 0.43, 95% CI 0.33–0.56, *p* < 0.001). Other independent adverse prognostic factors included AFP >20 ng/mL (HR = 1.39, 95% CI 1.09–1.76, *p* = 0.007), Hb<110 g/L (HR = 1.70, 95% CI 1.23–2.35, *p* = 0.001), and AST >40 U/L (HR = 1.57, 95% CI 1.24–2.00, *p* < 0.001). Male sex was associated with a lower risk of death (HR = 0.73, 95% CI 0.54–0.98, *p* = 0.034). BCLC stage was not statistically significant (HR = 1.22, 95% CI 0.96–1.54, *p* = 0.106). In the sensitivity model (replacing BCLC stage with tumor size, PVTT, and extrahepatic metastasis; Figure S1B), the cocktail regimen remained independently protective (adjusted HR = 0.40, 95% CI 0.31–0.53, *p* < 0.001). Together, these results confirm the cocktail regimen as a robust independent protective factor for overall survival in the pre‐matching cohort.

#### 2.2.2 Survival Outcomes After PSM

However, the imbalanced distribution of covariate factors before PSM may have affected the assessment of the relationship between patient survival and the effect of TCC treatment. Therefore, an analysis after PSM was conducted. After PSM, 67 patients in the cocktail group and 185 in the control group had died at the final follow‐up. The median follow‐up after matching was 80 months (range 4–103) in the cocktail group and 68 months (range 1–102) in the control group, calculated by the reverse Kaplan–Meier method (log‐rank *p* = 0.089). Overall survival was compared using Kaplan–Meier curves and the log‐rank test. The cocktail group had significantly longer median overall survival (OS) than the control group: 42.5 months (95% CI 31.0–not reached) vs. 12.5 months (95% CI 10.0–19.0); log‐rank *p* < 0.001 (Figure 3B and Table 2). The 1‐, 3‐, and 5‐year survival rates were 50.0% (95% CI: 43.9–56.9), 28.5% (95% CI: 23.2–35.0), and 19.8% (95% CI: 15.2–25.7) in the Control group, and 78.1% (95% CI: 70.8–86.0), 51.8% (95% CI: 43.3–61.8), and 43.9% (95% CI: 35.6–54.0) in the Cocktail group, respectively.

After PSM, all baseline covariates were well balanced (absolute SMD < 0.1 for all variables); therefore, multivariable Cox regression was not considered the primary analytical method. The hazard ratio (HR) for the cocktail group versus the control group was estimated using a treatment‐only Cox proportional hazards model (i.e., a univariable Cox model with treatment group as the only covariate). The cocktail regimen was associated with a significantly lower risk of death (HR = 0.54, 95% CI 0.40–0.75, *p* < 0.001; Figure 3B). As an exploratory analysis, we also performed a multivariable Cox regression as supportive evidence. The Cocktail regimen remained an independent protective factor (HR = 0.50, 95% CI 0.37–0.69, *p* < 0.001, Table S2).

Sensitivity analyses were performed to assess the robustness of the treatment effect on overall survival. In a 1:1 propensity score‐matched cohort (119 pairs; *n* = 238), the cocktail regimen remained associated with improved overall survival in a univariable Cox model with treatment group as the only covariate (HR = 0.53, 95% CI 0.386–0.716; *p* < 0.001). In the inverse probability of treatment weighting (IPTW) analysis of the full pre‐matching cohort (*n* = 433), an IPTW‐weighted multivariable Cox model adjusted for age, sex, ECOG performance status, Child–Pugh grade, BCLC stage, AFP, hemoglobin, AST, tumor size, PVTT, and extrahepatic metastasis also showed a consistent protective effect (adjusted HR = 0.41, 95% CI 0.304–0.559; *p* < 0.001). Both sensitivity analyses yielded results consistent with the primary post‐PSM analysis (1:2 matched cohort, treatment‐only Cox: HR = 0.54, 95% CI 0.40–0.75; *p* < 0.001), supporting the robustness of the observed survival benefit.

#### 2.2.3 Exploratory Subgroup Analyses

Exploratory subgroup and interaction analyses were performed using pre‐PSM data with multivariable Cox models including treatment‐by‐subgroup interaction terms, adjusted for age, sex, ECOG performance status, Child–Pugh grade, BCLC stage, AFP, tumor size, PVTT, extrahepatic metastasis, hemoglobin, AST (Figure S2). These analyses were considered hypothesis‐generating, and no correction for multiple testing was applied. Significant interactions were observed between treatment and tumor size (*p* for interaction = 0.004), AFP level (*p* for interaction = 0.011), and Hb level (*p* for interaction = 0.002). The survival benefit of the cocktail regimen was more pronounced in patients with tumor size >50 mm (HR = 0.34, 95% CI: 0.24–0.48, *p* < 0.001), AFP >20 ng/mL (HR = 0.36, 95% CI: 0.26–0.51, *p* < 0.001), and Hb ≥110 g/L (HR = 0.42, 95% CI: 0.31–0.55, *p* < 0.001). In contrast, no significant benefit was observed in patients with Hb <110 g/L (*p* = 0.434), and the effect was attenuated in those with AFP ≤20 ng/mL (*p* = 0.080) or tumor size ≤50 mm (*p* = 0.037).

#### 2.2.4 Tumor Response

In the pre‑matching cohort, the objective response rate (ORR) was 29.4% (95% CI 21.4–38.5) in the cocktail group and 24.2% (95% CI 19.6–29.3) in the control group, with no significant difference between the two groups (*p* = 0.270). However, the disease control rate (DCR) was significantly higher in the cocktail group than in the control group (79.0% [95% CI 70.6–85.9] vs. 61.5% [95% CI 55.8–66.9], *p* < 0.001, Table 3 and Figure 3C). In the cocktail group, complete response (CR) was observed in 10 patients (8.4%), partial response (PR) in 25 (21.0%), stable disease (SD) in 59 (49.6%), and progressive disease (PD) in 25 (21.0%). In the control group, CR occurred in 10 patients (3.2%), PR in 66 (21.0%), SD in 117 (37.3%), and PD in 121 (38.5%). The distribution of CR/PR/SD/PD differed significantly between groups (*p* = 0.001) (Table 3 **and** Figure 3D).

**TABLE 3:** Tumor response analysis comparing the control and cocktail groups before and after PSM.

Tumor response	Control	Cocktail	*p*‐value
Before PSM response	Distribution *p*‐value		0.001^*^
Objective response rate (95% CI)	24.2% (19.6–29.3)	29.4% (21.4–38.5)	0.270
Disease control rate (95% CI)	61.5% (55.8–66.9)	79.0% (70.6–85.9)	<0.001^*^
Complete response, *N* (%)	10 (3.2)	10 (8.4)	
Partial response, *N* (%)	66 (21.0)	25 (21.0)	
Stable disease, *N* (%)	117 (37.3)	59 (49.6)	
Progressive disease, *N* (%)	121 (38.5)	25 (21.0)	
After PSM response	Distribution *p*‐value		0.006^*^
Objective response rate (95% CI)	21.1 (15.9–26.9)	29.8 (21.6–39.1)	0.081
Disease control rate (95% CI)	61.8 (55.2–68.2)	79.8 (71.3–86.8)	<0.001^*^
Complete response, *N* (%)	10 (4.4)	10 (8.8)	
Partial response, *N* (%)	38 (16.7)	24 (21.1)	
Stable disease, *N* (%)	93 (40.8)	57 (50)	
Progressive disease, *N* (%)	87 (38.2)	23 (20.2)	

* *p*‐values <0.05 were considered to be statistically significant.

Abbreviations: PSM, propensity score matching; CI, confidence intervals.

After PSM, the ORR was 29.8% (95% CI 21.6–39.1) in the cocktail group and 21.1% (95% CI 15.9–26.9) in the control group (*p* = 0.081). The DCR remained significantly higher in the cocktail group (79.8% [95% CI 71.3–86.8] vs. 61.8% [95% CI 55.2–68.2], *p* < 0.001, Table 3 and Figure 3C). In the matched cocktail group, there were 10 patients (8.8%) with CR, 24 (21.1%) with PR, 57 (50%) with SD, and 23 (20.2%) with PD. In the matched control group, there were 10 patients (4.4%) with CR, 38 (16.7%) with PR, 93 (40.8%) with SD, and 87 (38.2%) with PD. The distribution of CR/PR/SD/PD differed significantly between groups (*p* = 0.006) (Table 3 and Figure 3D)

These findings indicate that the cocktail regimen achieved significantly better disease control than the control group in both the pre‑ and post‑matching cohorts, and also showed a superior objective response rate in the matched cohort. Post‐PSM response evaluation demonstrated significantly better tumor control in the cocktail group (Table 3 and Figure 3C, D). Representative image change of CR and PR are shown in Figure 4.

**FIGURE 4:**
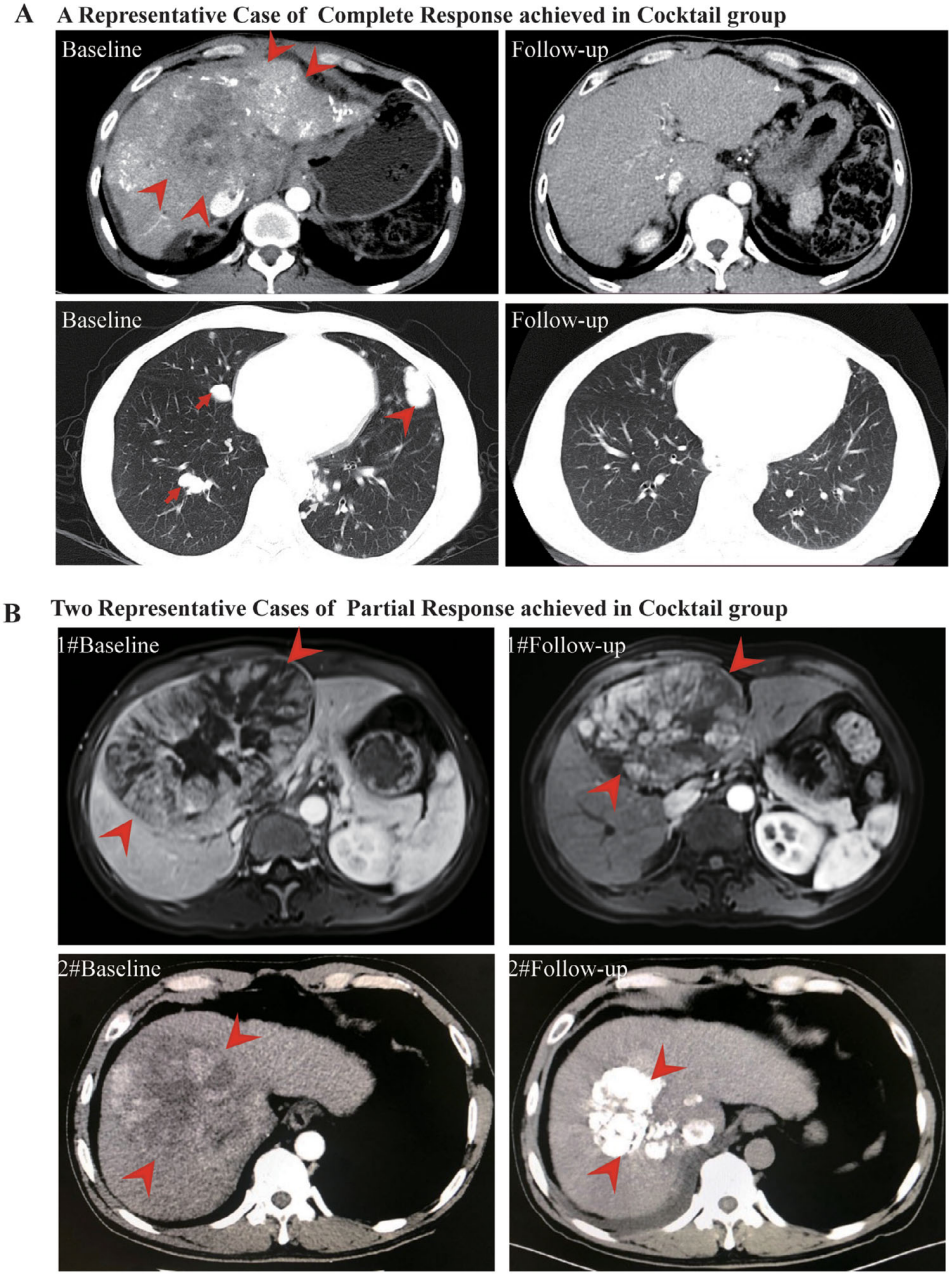
Representative radiological responses to cocktail therapy evaluated according to mRECIST criteria. Radiological assessment of intrahepatic and extrahepatic tumor response following combination therapy. **(A)** Contrast‐enhanced CT images of a patient with advanced HCC and pulmonary metastases before and after treatment. Baseline imaging demonstrated a large intrahepatic tumor with multiple satellite nodules and concomitant lung metastases. Follow‐up imaging after 6 months of therapy showed complete disappearance of both intrahepatic and pulmonary lesions, consistent with complete response according to mRECIST criteria. **(B)** Two representative cases illustrating marked regression of intrahepatic tumors after sequential TACE combined with cocktail therapy. Baseline arterial‐phase imaging revealed large enhancing hepatic masses. Follow‐up imaging demonstrated substantial reduction of viable enhancing tumor components, meeting criteria for partial response based on mRECIST. Arrows indicate target lesions.

### 2.3 Safety Analysis

The incidence of skin rash (*p <* 0.001) and pruritus (*p <* 0.001) was increased in the cocktail group. A total of 5 (5.5%) patients had thalidomide dose reductions due to grade 3 AEs. No life‐threatening AEs (grade 4 or 5) were detected in the cocktail and control groups. Most of the AEs were mild and self‐limiting. Moreover, other AEs including fatigue (*p* = 0.004), diarrhea (*p* = 0.031), and vomiting (*p* = 0.040) were significantly less common in the cocktail group (Table 4).

**TABLE 4:** Logistic models for toxicity in a well‐balanced cohort.

AEs, *N* (%)	Control	Cocktail	*p*‐value
Skin rash	2 (1.1)	29 (32.2)	<0.001^*^
Constipation	59 (32.8)	33 (36.7)	0.618
Fatigue	69 (38.3)	18 (20.0)	0.004^*^
Pruritus	2 (1.1)	13 (14.4)	<0.001^*^
Diarrhea	36 (20.0)	8 (8.9)	0.031^*^
Vomiting	40 (18.3)	10 (11.1)	0.040^*^

* *p*‐values <0.05 were considered to be statistically significant.

Abbreviations: AEs, adverse events; CI, confidence intervals; OR, odds ratio.

## 3 Discussion

Patients with unresectable HCC still have a poor prognosis and various combination treatments have been proposed and applied in clinical practice. Our multicenter retrospective cohort study found that compared with TACE alone, TCC combined with TACE significantly improved the OS rates as well as ORR and DCR, with no serious AEs detected during treatment or follow‐up. Taken together, these findings suggest that TCC may be a feasible adjunct to TACE in selected patients with unresectable HCC.

Although combining TACE with systemic therapies represents an important area of investigation, current evidence suggests only modest clinical benefits. For instance, a large retrospective study (CHANCE001), including 826 HCC patients receiving either TACE plus PD‐(L)1 blockades and molecular targeted therapy (*n*  =  376) or TACE monotherapy (*n*  =  450), demonstrated OS was slightly prolonged in the combination group (median OS, 19.2 months vs. 15.7 months, *p* =  0.001) [16]. In the IMbrave150 trial, analyses (using HCC RECIST criteria) that compared a combination treatment regimen of atezolizumab plus bevacizumab to sorafenib treatment alone found an ORR of 27.3% vs. 11.9% (5.5% vs. 0% CR, 21.8% vs. 11.9% PR), with SD in 46.3% vs. 43.4% of patients and PD in 19.6% vs. 24.5% [10]. Recently, the Phase III LEAP‐012 trial demonstrated that the addition of lenvatinib plus pembrolizumab to TACE significantly improved progression‐free survival and ORR compared with TACE alone, yet the survival benefit was modest and grade 3–4 adverse events were notable [27]. Moreover, a median ORR of 16% was reported in the KEYNOTE‐224 Phase II clinical trial in 51 HCC patients treated with pembrolizumab [28], and the ORR was 15% vs. 7% in a randomized Phase III trial that compared nivolumab to sorafenib as first‐line treatment of advanced HCC [29]. In summary, the above findings from contemporary trials indicate that even in the era of immuno‐oncology and targeted therapy, TACE‐based combinations still require further optimization. In contrast, although the components of the TCC cocktail are “older” drugs, both our present multicenter retrospective study and our recently published prospective randomized controlled trial (RCT) have demonstrated clear antitumor activity. In the current study, after propensity score matching, the TCC plus TACE group achieved a median overall survival (OS) of 42.5 months, significantly longer than the 12.5 months in the TACE‐alone group (HR = 0.54, 95% CI 0.40–0.75, *p* < 0.001). This real‐world finding corroborates our prospective RCT, which reported a median OS of 29.0 months, a median progression‐free survival of 16.0 months, and a 3‐year OS rate of 37.7% [19]. Collectively, these results suggest that the TCC cocktail confers a substantial survival benefit when added to TACE. Of note, patients in the present study were enrolled between 2014 and 2018, a period during which TACE remained the standard treatment for advanced HCC in China—particularly for patients with preserved liver function and predominantly intrahepatic tumor burden. This practice was consistent with the China Liver Cancer (CNLC) staging system and the contemporary expert consensus at that time [30]. Even in the presence of extrahepatic metastases, effective control of intrahepatic lesions could still provide a survival benefit, because intrahepatic progression and subsequent liver failure remain the leading causes of death in advanced HCC. Given the differences in study design, patient populations, treatment strategies, and follow‐up durations across these trials, direct numerical comparisons should be interpreted with caution. Therefore, the observed differences do not necessarily imply superiority or inferiority of any particular regimen, but rather provide contextual background for understanding the potential clinical activity of TACE combined with the TCC cocktail. Although the TCC cocktail improved overall survival in the overall population, the benefit was not uniform across all patients. Exploratory subgroup analysis revealed a significant interaction between treatment benefit and baseline hemoglobin level (interaction *p*  = 0.002): patients with baseline hemoglobin <110 g/L derived no survival advantage from the TCC regimen (HR = 0.85, 95% CI 0.56–1.29, *p* = 0.434), whereas those with hemoglobin ≥110 g/L experienced a marked benefit (HR = 0.42, 95% CI 0.31–0.55, *p* < 0.001). Several mechanisms may explain this finding. Low hemoglobin often reflects poor nutritional status, impaired liver function reserve, or bone marrow suppression—all of which can attenuate the efficacy of chemotherapy‐based regimens. Moreover, anemia itself can induce tumor hypoxia and thereby promote chemoresistance. From a clinical decision‐making perspective, this hypothesis‐generating finding suggests that baseline hemoglobin level may serve as a potential biomarker for selecting patients most likely to benefit from the TCC regimen. Specifically, patients with hemoglobin ≥110 g/L are more likely to benefit, whereas those with severe anemia should be treated with caution or receive anemia correction first. Prospective validation of this hypothesis is warranted.

The results of the present study suggested that the combination of the TCC with TACE may represent a potentially favorable approach for treating unresectable HCC. The observed longer survival coupled with a reduced incidence of AEs suggested that the cocktail combined with TACE had a synergistic effect in our study. The mechanisms of synergistic anti‐tumor effect include the following aspects. First, in recent years, studies have found that thalidomide not only inhibits the expression of the angiogenic agent vascular endothelial growth factor (VEGF), which is upregulated in tumor cells that survive ischemia [31, 32], but also reverses the effects of growth differentiation factor 15, which was found to be upregulated in chemotherapy‐damaged HCC cells [33]. Thus, TACE combined with thalidomide prevents tumor angiogenesis and inhibits the recurrence of liver cancer after TACE. Second, the cocktail also induces cell cycle arrest in different phases. It has been reported that the progression of the cell cycle can be blocked by thalidomide from the G0 to G1 phase [34] and by cantharidine from the G1 to S phase [35]. Also, carmofur interferes with DNA synthesis in tumor cells and thus affects the cell cycle [36]. Moreover, thalidomide induces T‐cell co‐stimulation and modulates cytokine expression to augment anti‐tumor immunity [37, 38]. 5‐fluorouracil slightly increased circulating Tregs at medium doses, and increased circulating NKs at low doses [39] and downregulated the signaling proteins of the nuclear transcription factor κB (NF‐κB) pathway [40]. It is noteworthy that 5‐fluorouracil resistance has become a major obstacle to its clinical use in HCC patients, but the compound cantharidin capsule has shown the effect of reversing drug resistance during treatment [41]. Furthermore, our recent research provides evidence for the immunotherapeutic mechanism of TCC that can reverse tumor immune escape by targeting SAMD4B‐APOA2‐PD‐L1 axis [26].

The TCC regimen demonstrated a favorable safety profile in this study. No treatment‐related grade 4–5 life‐threatening adverse events (AEs) were observed in either group, and the cocktail group had significantly lower incidences of fatigue, diarrhea, and vomiting compared with the control group. This safety advantage can be largely attributed to the metronomic administration of carmofur. Frequent low‐dose dosing without prolonged drug‐free intervals balances antitumor activity with tolerability [42]. As a time‐dependent fluoropyrimidine, frequent low‐dose administration may also be pharmacokinetically advantageous, a strategy that has shown potential clinical benefit in other malignancies, including metastatic nasopharyngeal carcinoma [43, 44]. Emerging evidence suggests that low‐dose chemotherapy may induce tumor cell senescence, thereby suppressing tumor proliferation and limiting additional genomic instability [45]. Compared with previous reports, the reduced‐dose carmofur regimen used in our study appeared to be associated with fewer treatment‐related toxicities and no serious adverse events [46]. Furthermore, the lower rates of vomiting and diarrhea in the cocktail group support the hypothesis that the constipating effect of thalidomide counteracts the diarrheal effects caused by CMC and carmofur. Dose reduction of thalidomide and carmofur also contributed to minimizing AEs; for example, although thalidomide may increase thrombotic risk in a cancer‐related hypercoagulable state [47, 48], no thrombotic events were observed in this study.

We acknowledge the inherent limitations of retrospective safety data collection (recall bias, incomplete documentation), and mild‐to‐moderate AEs may be underreported. However, no treatment‐related life‐threatening (grade 4–5) adverse events were observed in either group throughout the entire retrospective cohort. It was a key finding consistent with our prospective randomized controlled trial, in which the grade 3–4 adverse event rate in the TACE+TCC group was only 11.1%, virtually identical to the TACE‐alone group (8.3%) [19]. This contrasts sharply with IO/TKI combinations, where grade 3–4 AE rates typically exceed 30% (IMbrave150: 56.5%; CHANCE001 combination group: 16.7% vs. TACE alone 8.2%). The combination therapy has been proven to be effective in countering the AEs produced by each medication present in TCC.

Beyond efficacy and safety, the TCC regimen offers substantial advantages in cost and convenience. First‐line preferred regimens including atezolizumab + bevacizumab (T+A) are too expensive to be widely used in clinical practice in China and many other low‐ and middle‐income countries. In contrast, TCC improved cancer control while maintaining a minimum cost of treatment, about 1% of the cost of “T+A” therapy (see Table S3). Oral administration reduces the frequency of hospital visits, and the lower incidence of AEs is expected to improve patient compliance. Therefore, the TCC regimen provides a low‐toxicity, affordable, and convenient alternative for patients with compromised liver function, poor performance status, intolerance to intensive immunotherapy, or limited access to expensive therapies—a treatment gap increasingly recognized in global oncology discourse [42, 49]. Nevertheless, before wider clinical adoption, prospective head‐to‐head comparisons between the TCC regimen and contemporary standard regimens are needed to establish its relative efficacy, safety, and generalizability.

Several limitations of this study should be acknowledged when interpreting the findings. First, the retrospective, non‐randomized design inherently carries potential selection bias and unmeasured confounding, as propensity score matching can only balance observed covariates, it cannot eliminate bias from unmeasured factors such as detailed performance status, comorbidities, subsequent therapies, or physician treatment preferences. Consequently, residual confounding—including the possibility that fitter patients were more likely to receive the TCC regimen—cannot be ruled out, and our findings should be interpreted as associations rather than causal effects. Second, the study period (2014–2018) predates the widespread adoption of modern immunotherapy and targeted therapy combinations for HCC. Thus, the results may not fully reflect the outcomes expected in the current era where such regimens are standard first‐line options. Third, the specific TCC cocktail (thalidomide, carmofur, and compound mylabris capsule) is not guideline‐recommended for HCC in most countries and faces significant regulatory and acceptability barriers outside China, limiting its generalizability to Western populations and other healthcare settings. Fourth, safety data collection in retrospective studies is inherently incomplete. Although we systematically reviewed medical records, mild‐to‐moderate adverse events (especially grade 1–2) may have been underreported. A comprehensive safety assessment would require prospective data collection with standardized toxicity grading. Fifth, tumor response was primarily assessed according to mRECIST criteria, which focus on viable intrahepatic lesions. Extrahepatic lesions were not systematically evaluated, and information on post‐progression systemic therapies was not collected. As subsequent treatments may affect overall survival, their potential impact could not be assessed. Sixth, the absence of direct comparison with contemporary standard systemic therapies (e.g., sorafenib, lenvatinib, atezolizumab‐bevacizumab) precludes determination of the relative efficacy or non‐inferiority of the TCC regimen. Seventh, the study population was predominantly Chinese with HBV‐related HCC. Therefore, external validation in other ethnicities and etiologies is warranted. In addition, because some participating centers contributed a small number of patients and the distribution of patients across centers was uneven, including center as a categorical variable in the propensity score matching (PSM) model would have led to sparse strata, unstable propensity score estimation, and substantial loss of matched patients. To avoid these consequences, center was not included in the PSM model; this may have left residual center‐related confounding that could not be fully excluded. Despite these limitations, the consistency between our retrospective findings and a previously published prospective randomized controlled trial partially mitigates concerns regarding bias. Nonetheless, the results should be considered hypothesis‐generating, and future prospective randomized trials directly comparing the TCC cocktail with modern combination regimens are needed to establish its role in the current treatment landscape.

## 4 Conclusion

In conclusion, this multicenter retrospective cohort study suggests that the addition of thalidomide‐based cocktail to TACE may improve survival outcomes for patients with unresectable HCC, with a manageable safety profile. These real‐world findings corroborate the results of our prospective randomized trial and demonstrate that TACE combined with TCC can achieve meaningful survival benefits in routine clinical practice. Given the inherent limitations of retrospective research, these results should be considered hypothesis‐generating. The TCC regimen may be particularly suitable for patients who cannot tolerate intensive IO/TKI therapy or who lack access to these expensive treatments. Future prospective randomized trials comparing TCC with modern systemic regimens (such as TACE combined with IO/TKI) are warranted to definitively establish its role in the current treatment landscape.

## 5 Methods

### 5.1 Patients

A total of 545 unresectable HCC patients were screened who met the diagnostic criteria according to the Guidelines for Diagnosis and Treatment of Primary Liver Cancer in China [5] and underwent conventional TACE. After applying the inclusion and exclusion criteria, 433 patients were enrolled from Zhongshan Hospital, Fudan University (*n* = 206); Minhang Hospital, Fudan University (*n* = 8); Kecheng People's Hospital (*n* = 8); and The First Affiliated Hospital of Wenzhou Medical University (*n* = 211) in China between March 2014 and May 2018. After applying the inclusion and exclusion criteria, 433 patients were enrolled and followed up for clinical outcomes until July 2023. Patients were eligible if they: (1) were adult patients (≥18 years old), (2) had unresectable HCC diagnosed according to the Chinese guidelines, (3) received initial conventional TACE, (4) had complete medical records available for follow‐up. The exclusion criteria were patients with: (1) missing or incomplete medical records, (2) previous or synchronous anti‐tumor treatment, such as radiofrequency ablation (RFA) or systemic chemotherapy; (3) poor liver function (Child–Pugh class C); and (4) other life‐threatening diseases. Patients were divided into the cocktail group (TCC + TACE) and the control group (TACE alone) according to whether they received the TCC cocktail in addition to TACE. The study was approved by research ethics committees of the participating hospital (B2025‐756) and registered at ClinicalTrials.gov (NCT07321613).

### 5.2 Management of the Patients

Data were collected for each patient before undergoing TACE, including age, sex, serum alpha‐fetoprotein (AFP) concentration, reports of blood routine and biochemical examination, hepatitis history, tumor size and number, extrahepatic metastasis, and the Eastern Cooperative Oncology Group (ECOG) performance status as well as BCLC stage [50]. All patients received initial treatment with TACE by well‐trained and experienced physicians. Briefly, after the femoral artery was punctured using Seldinger technique and the identification of the hepatic artery, a mixture of pirarubicin 20–50 mg, oxaliplatin 50–100 mg, and lipiodol 5–25 mL (the quantity administered depended on the tumor size) was utilized to produce embolization. Hypervascular tumors were also embolized with Gelatin foam [51]. Repeated TACE procedures were carried out for patients with active tumor as indicated by follow‐up contrast‐enhanced CT/MRI images. Tumor response was assessed at the 6‐month follow‐up based on contrast‐enhanced CT/MRI scans using the modified Response Evaluation Criteria in Solid Tumors for hepatocellular carcinoma (mRECIST) [52]. According to mRECIST, responses for target lesions were defined as follows: complete response (CR), disappearance of any intratumoral arterial enhancement in all target lesions; partial response (PR), at least a 30% decrease in the sum of diameters of viable (arterially enhancing) tumor; progressive disease (PD), at least a 20% increase in the sum of diameters of viable tumor or the appearance of a new lesion; and stable disease (SD), which met the criteria for neither PR nor PD. Viable intrahepatic HCC target lesions were evaluated according to mRECIST, based on arterial enhancement. Extrahepatic lesions were not included in the measurement of viable intrahepatic target lesions; however, baseline extrahepatic lesions, new extrahepatic lesions, and non‐target lesions were reviewed during follow‐up and considered in the overall response assessment, especially for determining progressive disease. Thus, new extrahepatic lesions or unequivocal progression of existing extrahepatic disease was classified as PD. All responses were determined in comparison to the baseline assessment.

Patients in the cocktail group initially took the combined oral medications daily, which consisted of thalidomide tablets (Changzhou Pharmaceutical Factory, 50 mg qn), carmofur tablets (Qilu Pharmaceutical, 100 mg, tid), and CMC (Guizhou Yibai Pharmaceutical Co., Ltd, 750 mg, bid) within 30 days of the first TACE procedure, with continuous treatment for a minimum of 1 month. Once the CR was reached, patients stopped taking carmofur tablets, while the original doses of oral thalidomide and cantharidin sodium capsules were maintained if tolerated by the patients. OS was evaluated as the interval from the date of a patient's first TACE treatment until the date of death or the last follow‐up. AEs were assessed and graded using Common Terminology Criteria for Adverse Events Version 4.0 (CTCAE version 4.0.3).

### 5.3 Statistical Analysis and Propensity Matching

Comparison of the baseline characteristics of patients was made using a chi‐squared or Fisher's exact tests for categorical variables, or the Wilcoxon rank test for continuous variables, to look for differences as appropriate. Several clinically relevant covariates were categorized based on established clinical cutoffs, whereas tumor diameter, log‐transformed AFP, platelet count, hemoglobin, and AST/ALT ratio were entered as continuous variables where appropriate. Laboratory results of blood routine tests were classified according to the lower limit of normal for blood routine parameters and the upper limit of normal for biochemical parameters, with an AFP cutoff of 20 ng/mL [53]. The 50‐mm cutoff of tumor diameter was used only for exploratory subgroup visualization because it is commonly used as a clinically interpretable threshold in HCC staging and prognostic assessment. TACE frequency was considered a post‐treatment variable that may lie on the causal pathway between treatment and survival. Therefore, it was not included in the propensity score model, primary Cox regression, subgroup analyses, or sensitivity analyses. The primary analysis focused on estimating the effect of treatment assignment. Propensity score matching (PSM) was then conducted based on patients’ baseline clinical characteristics and tumor status. Covariates included: age (>60 vs.≤60 years), sex, hepatitis history, Child‐Pugh class (B vs. A), ECOG performance status (1–2 vs. 0), BCLC stage (C vs. B), log‐transformed alpha‐fetoprotein (logAFP), tumor number (>3 vs. ≤3), maximum tumor diameter (continuous, mm), portal vein tumor thrombosis (PVTT, yes/no), extrahepatic metastasis (yes/no), baseline hemoglobin (g/L), platelet count (×10^9^/L), white blood cell count (≤4 vs. >4×10^9^/L), and AST/ALT ratio. Because ALT and AST were highly correlated (Pearson r = 0.76), the AST/ALT ratio was used instead of including both variables separately to avoid multicollinearity. The matching approach was set as the nearest neighbor algorithm with a matching ratio of 1:2, a caliper of 0.2 times the standard deviation of the logit‐transformed propensity score, and discarding samples outside the common support region using the MatchIt package in R version 4.3.1 [54].

For the pre‐PSM cohort, multivariable Cox proportional hazards regression was performed to evaluate the association between treatment group and overall survival. To avoid data‐driven variable selection and reduce the risk of overfitting, covariates were selected a priori based on clinical relevance rather than solely on statistical significance in univariable analyses. For example, age and sex were forced into the model regardless of their univariate significance, and other pre‑specified clinically relevant covariates included ECOG performance status, Child–Pugh class, AFP level, BCLC stage, and tumor burden variables (tumor size, PVTT, and extrahepatic metastasis). After PSM, survival outcomes were compared primarily using Kaplan–Meier analysis and the log‐rank test in the matched cohort. A Cox proportional hazards model including treatment group only was used to estimate the hazard ratio after matching.

To assess covariate balance before and after matching, we used standardized mean differences (SMDs) instead of *p*‐values. For binary variables, SMD was calculated as the difference in proportions divided by the pooled standard deviation: SMD = (*p*1 – *p*2) / sqrt(*p*_pooled * (1 – *p*_pooled));where: *p*1 = proportion of the event in the treatment group (cocktail), *p*2 = proportion of the event in the control group (control), *p*_pooled = (*p*1 + *p*2) / 2. For the post‐matching analysis, the SMDs were weighted using the matching weights generated by the 1:2 matching procedure (implemented via the survey package in R). An SMD absolute value < 0.1 was considered good balance, < 0.2 acceptable balance, and > 0.2 meaningful imbalance.

The OS curves were evaluated using Kaplan‒Meier analysis. The log‐rank test, the hazard ratio (HR), and 95% confidence intervals (CIs) were calculated using the Cox method. Differences in the distribution of treatment response (CR, PR, SD, PD) between groups were compared using the chi‐square test; Fisher's exact test was used when expected frequencies were less than 5. Comparisons of objective response rate (ORR) and disease control rate (DCR) were also performed using the chi‐square test or Fisher's exact test. All tests were two‐sided, and *p* < 0.05 was considered statistically significant. Logistic regression models were used to compare AEs. All statistical analyses were performed using R version 4.3.1. A *p*‐value *<*0.05 was deemed to be a statistically significant finding.

## Author Contributions

Study concept and design: Jinglin Xia, Jinzhi Xu, Jian Zhang, and Jie Li. Data collection and follow‐up: Jinzhi Xu, Jian Zhang, Jie Li, Yifan Kong, Tao Ye, Yongqiang Liu, Biwei Yang, Zhiying Zhao, Peixin Huang, Yi Chen, Ningling Ge, Guoqing Zhu, Xin Yin, and Wenhao Hu. Statistical analysis: Jinzhi Xu, Jie Li, Yifan Kong, and Li Song. Manuscript drafting and revision: Jinzhi Xu, Jian Zhang, Jie Li, Faiz Wali, Matteo Jiang, Li Song, and Jinglin Xia. All authors have read and agreed to the published version of the manuscript.

## Funding

Financial support was provided by the National Natural Science Foundation of China (81772590, 81572395).

## Ethic Statements

The study protocol conformed to the ethical guidelines of the 1975 Declaration of Helsinki and was approved by the Ethics Committee of Zhongshan Hospital, Fudan University, Shanghai, China (B2025‐756). This retrospective, multicenter cohort study was registered at ClinicalTrials.gov (NCT07321613). Patients’ informed consent was waived or not required, as this is a retrospective study, and the analysis used anonymous clinical data that were obtained after the patient or their guardians had agreed to treatment with written consent.

## Conflicts of Interest

The authors have no conflicts of interest to declare.

## Supporting information




**Supporting File 1**: mco271116‐sup‐0001‐SuppMat.docx.

## Data Availability

Research data are not publicly available on legal or ethical grounds. Further inquiries can be directed to the corresponding author
