# Supplementary material for: Enhanced Efficacy of Thalidomide‐Based Cocktail With Transarterial Chemoembolization in the Management of Unresectable Hepatocellular Carcinoma: A Multi‐Center Cohort Study
Source: MedComm (2020). 2026 Oct 31;7(11):e71116. doi: 10.1002/mco2.71116 (PMC13722950; doi:10.1002/mco2.71116)
Supplement: Supplementary file 1 [file MCO2-7-e71116-s001.docx]

***Research Article***

***Enhanced Efficacy of Thalidomide-Based Cocktail with Transarterial Chemoembolization in the management of Unresectable Hepatocellular Carcinoma: A Multi-Center Cohort Study***

Jinzhi Xu^1,6#^, Li Song^1,7#^, Yifan Kong^2#^, Jie Li^1#^, Jian Zhang^1#^, Tao Ye^3^, Yongqiang Liu^4^, Xin Yin^1^, Biwei Yang^1^, Zhiying Zhao^1^, Peixin Huang^1^, Yi Chen^1^, Matteo Jiang^1^, Ningling Ge^1^, Faiz Wali^2^, Wenhao Hu^5^, Guoqing Zhu^5^, Jinglin Xia^1,2*^

^1^National Medical Center and National Clinical Research Center for Interventional Medicine, Liver Cancer Institute, Zhongshan Hospital, Fudan University, 180 Fenglin Road, Shanghai 200032, China

^2^Zhejiang Key Laboratory of Intelligent Cancer Biomarker Discovery and Translation, The First Affiliated Hospital of Wenzhou Medical University, Wenzhou 325035, Zhejiang, China

^3^Department of Oncology, Minhang Hospital, Fudan University, Shanghai 200032, China

^4^Department of Radiology, Kecheng People's Hospital, Quzhou 324000, Zhejiang, China

^5^Department of Interventional Radiology, The First Affiliated Hospital of Wenzhou Medical University, Wenzhou 325000, Zhejiang, China

^6^Department of Hematology, Shanghai Geriatric Medical Center, Zhongshan Hospital Fudan University Minhang Campus, Shanghai 200032, China.

^7^Department of Infectious Diseases, Shanghai Sixth People's Hospital Affiliated to Shanghai Jiao Tong University School of Medicine, Shanghai 200233, China

Short Title: TCC improves the efficacy of TACE for HCC

^#^ Contributed equally.

* Corresponding Author:

Jinglin Xia, MD, PhD, National Medical Center & National Clinical Research Center for Interventional Medicine; Liver Cancer Institute, Zhongshan Hospital, Fudan University, 180 Fenglin Road, Shanghai 200032, China;

E-mail address: xiajinglin@fudan.edu.cn.

Table S1. Cox Analysis of Clinical Characteristics Associated with Overall Survival before PSM.

| Variable | Level | Univariate Analysis | | Multivariate Analysis (main model) | |
| --- | --- | --- | --- | --- | --- |
|  |  | **HR (95%CI)** | ***p* value** | **HR (95%CI)** | ***p* value** |
| Cohort | **Cocktail vs Control** | 0.46 (0.35 - 0.60) | < 0.001* | 0.43 (0.33-0.56) | <0.001* |
| Age (y) | **>60 vs ≤60** | 1.28 (1.03 - 1.59) | 0.025* | 1.21 (0.97-1.52) | 0.092 |
| Sex | **Male vs Female** | 0.73 (0.56 - 0.95) | 0.021* | 0.73 (0.54-0.98) | 0.034 |
| Hepatitis history | **Yes vs No** | 0.92 (0.69 - 1.21) | 0.531 |  | NA |
| Child-Pugh | **B vs A** | 1.63 (1.19 - 2.22) | 0.002* | 1.22 (0.96-1.68) | 0.234 |
| ECOG PS | **1-2 vs 0** | 1.18 (0.95 - 1.46) | 0.139 | 1.17 (0.93-1.47) | 0.192 |
| BCLC | **C vs B** | 1.13 (0.91 - 1.40) | 0.269 | 1.22 (0.88-1.68) | 0.106 |
| AFP (ng/mL) | **>20 vs ≤20** | 1.26 (1.01 - 1.58) | 0.041* | 1.39 (1.09-1.76) | 0.007* |
| HB (g/L) | **<110 vs ≥110** | 1.87 (1.40 - 2.52) | < 0.001* | 1.70 (1.23-2.35) | 0.001* |
| WBC (×10^9/L) | **>4 vs ≤4** | 1.13 (0.88 - 1.44) | 0.334 |  | NA |
| PLT (×10^9/L) | **<100 vs ≥100** | 0.85 (0.66 - 1.09) | 0.196 |  | NA |
| ALT (U/L) | **>40 vs ≤40** | 1.06 (0.85 - 1.31) | 0.627 |  | NA |
| AST (U/L) | **>40 vs ≤40** | 1.44 (1.15 - 1.81) | 0.001* | 1.57 (1.24-2.00) | <0.001* |
| Number | **> 3 vs ≤ 3** | 1.04 (0.83 - 1.30) | 0.749 |  | NA |
| Size (mm) | **>50 vs ≤50** | 1.94 (1.55 - 2.42) | < 0.001* |  | NA |
| PVTT | **Yes vs No** | 1.41 (1.12 - 1.77) | 0.003* |  | NA |
| Metastasis | **Yes vs No** | 1.38 (1.11 - 1.73) | 0.004* |  | NA |

Note: Data are expressed as means ± SD or number (percentages). **p*-values <0.05 were considered to be statistically significant. Abbreviations: AFP, alpha fetoprotein; ALT, alanine aminotransferase; AST, aspartate aminotransferase; BCLC, Barcelona Clinic Liver Cancer; Hb, hemoglobin; PLT, platelet; PSM, propensity score matching; PVTT, portal vein tumor thrombus; WBC, white blood cell.

Table S2. Cox Analysis of Clinical Characteristics Associated with Overall Survival after PSM.

| Variable | Level | Univariate Analysis | | Multivariate Analysis | |
| --- | --- | --- | --- | --- | --- |
|  |  | **HR (95%CI)** | ***p* value** | **HR (95%CI)** | ***p* value** |
| Cohort | **Cocktail vs Control** | 0.54 (0.40 - 0.75) | < 0.001* | 0.50 (0.37-0.69) | <0.001* |
| Age (y) | **>60 vs ≤60** | 1.09 (0.83 - 1.45) | 0.529 |  | NA |
| Sex | **Male vs Female** | 0.76 (0.53 - 1.09) | 0.132 |  | NA |
| Hepatitis history | **Yes vs No** | 1.26 (0.83 - 1.92) | 0.282 |  | NA |
| Child-Pugh | **B vs A** | 1.26 (0.79 - 2.03) | 0.334 |  | NA |
| ECOG PS | **1-2 vs 0** | 1.28 (0.97 - 1.70) | 0.081 |  | NA |
| BCLC | **C vs B** | 1.53 (1.16 - 2.03) | 0.003* | 1.33 (0.83-2.13) | 0.242 |
| AFP (ng/mL) | **>20 vs ≤20** | 1.32 (0.98 - 1.77) | 0.069 |  | NA |
| HB (g/L) | **<110 vs ≥110** | 2.23 (1.49 - 3.34) | < 0.001* | 2.19 (1.44-3.33) | < 0.001* |
| WBC (×10^9/L) | **>4 vs ≤4** | 1.13 (0.83 - 1.55) | 0.430 |  | NA |
| PLT (×10^9/L) | **<100 vs ≥100** | 0.84 (0.61 - 1.15) | 0.278 |  | NA |
| ALT (U/L) | **>40 vs ≤40** | 1.05 (0.79 - 1.39) | 0.737 |  | NA |
| AST (U/L) | **>40 vs ≤40** | 1.46 (1.08 - 1.96) | 0.013* | 1.32 (0.96-1.81) | 0.082 |
| Number | **> 3 vs ≤ 3** | 1.13 (0.84 - 1.52) | 0.425 |  | NA |
| Size (mm) | **>50 vs ≤50** | 1.88 (1.41 - 2.50) | < 0.001* | 1.55 (1.12-2.15) | 0.008* |
| PVTT | **Yes vs No** | 1.71 (1.28 - 2.28) | < 0.001* | 0.96 (0.58-1.58) | 0.861 |
| Metastasis | **Yes vs No** | 1.63 (1.23 - 2.17) | 0.001* | 0.75 (0.29-1.94) | 0.551 |

Note: Data are expressed as means ± SD or number (percentages). **p*-values <0.05 were considered to be statistically significant. Abbreviations: AFP, alpha fetoprotein; ALT, alanine aminotransferase; AST, aspartate aminotransferase; BCLC, Barcelona Clinic Liver Cancer; Hb, hemoglobin; PLT, platelet; PSM, propensity score matching; PVTT, portal vein tumor thrombus; WBC, white blood cell.

TABLE S3. Cost of unresectable HCC patients.

| **Regents** | **Cocktail** | **Atezolizumab + Bevacizumab** | **Tremelimumab + Durvalumab** |
| --- | --- | --- | --- |
| **Cost** | ¥688 every 3 weeks | ¥46300 (60kg) every 3 weeks | ¥250,000 for the first time, and ¥55,000 every 3 weeks thereafter |

**Figure S1. Multivariable Cox regression analyses of overall survival in the pre-PSM cohort.**

(A) Main model including BCLC stage. (B) Sensitivity model replacing BCLC stage with tumor size, PVTT, and extrahepatic metastasis.

Hazard ratios (HRs) and 95% confidence intervals (CIs) were derived from multivariable Cox proportional hazards models. The model in (A) was adjusted for age, sex, ECOG performance status, Child–Pugh grade, BCLC stage, AFP, hemoglobin, and AST. The model in (B) was adjusted for age, sex, ECOG performance status, Child–Pugh grade, AFP, hemoglobin, AST, tumor size, PVTT, and extrahepatic metastasis. The vertical dashed line indicates an HR of 1. Orange squares indicate statistically significant variables (p < 0.05); gray squares indicate non-significant variables. Error bars represent 95% CIs. The numbers in parentheses indicate the number and percentage of patients with the corresponding risk factor. AFP, alpha-fetoprotein; AST, aspartate aminotransferase; BCLC, Barcelona Clinic Liver Cancer; CI, confidence interval; ECOG PS, Eastern Cooperative Oncology Group performance status; HR, hazard ratio; PVTT, portal vein tumor thrombus.

Figure S2. Forest plot of subgroup analyses evaluating the consistency of treatment benefit across predefined clinical variables.
